# Supplementary material for: Nonlinear dynamic characterization of two-dimensional materials
Source: Nat Commun. 2017 Nov 1;8:1253. doi: 10.1038/s41467-017-01351-4 (PMC5666000; doi:10.1038/s41467-017-01351-4)
Supplement: Supplementary file 1 — Supplementary Information [file 41467_2017_1351_MOESM1_ESM.pdf]

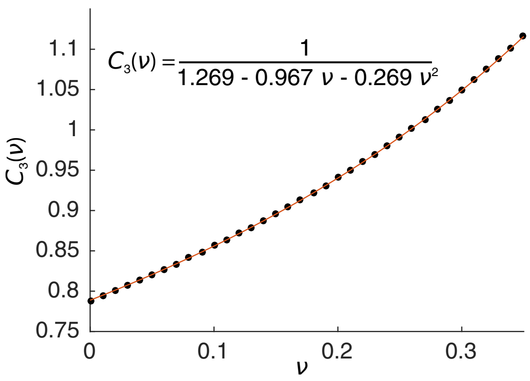

Supplementary Figure 1. **Numerical solutions for C_3 as a function of ν.** The red line is represents the corresponding fit.

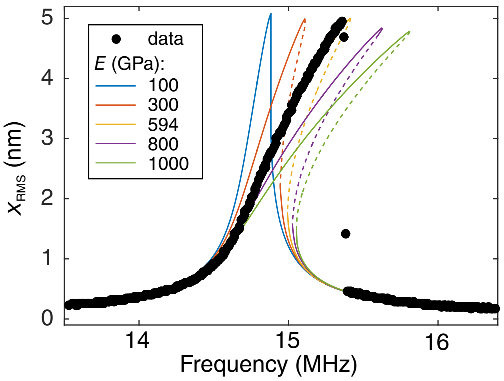

Supplementary Figure 2. **Sensitivity of the nonlinear response to the Young's modulus**. The measured trace (at $F_{\mathrm{RMS}} = 48$ pN) is represented by the black dots. The coloured lines represent the modelled response using fixed values for the damping and the driving force and varying values for the Young's modulus. The yellow line represents the modelled response using the fitted value for the Young's modulus ($E = 594$ GPa).

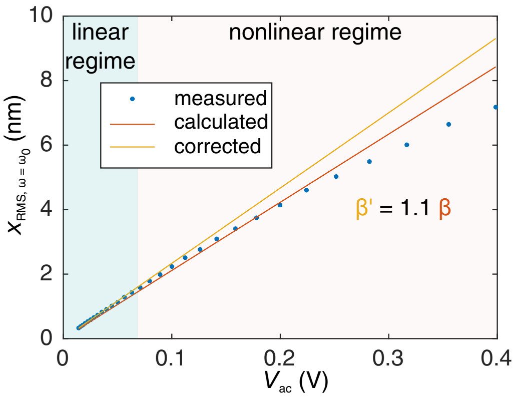

Supplementary Figure 3. **Force estimation and correction**. The dc voltage applied is $V_{\mathrm{dc}} = 1$ V. The blue dots represent the measured amplitude of a 8-nm thick FLG drum at resonance. The red curve is the calculated amplitude using Supplementary Equation (33) (in the linear regime) with the force calculated from Supplementary Equation (34). The yellow curve is the corrected force using Supplementary Equation (35). The error in the force estimation in this case is roughly 10 %.

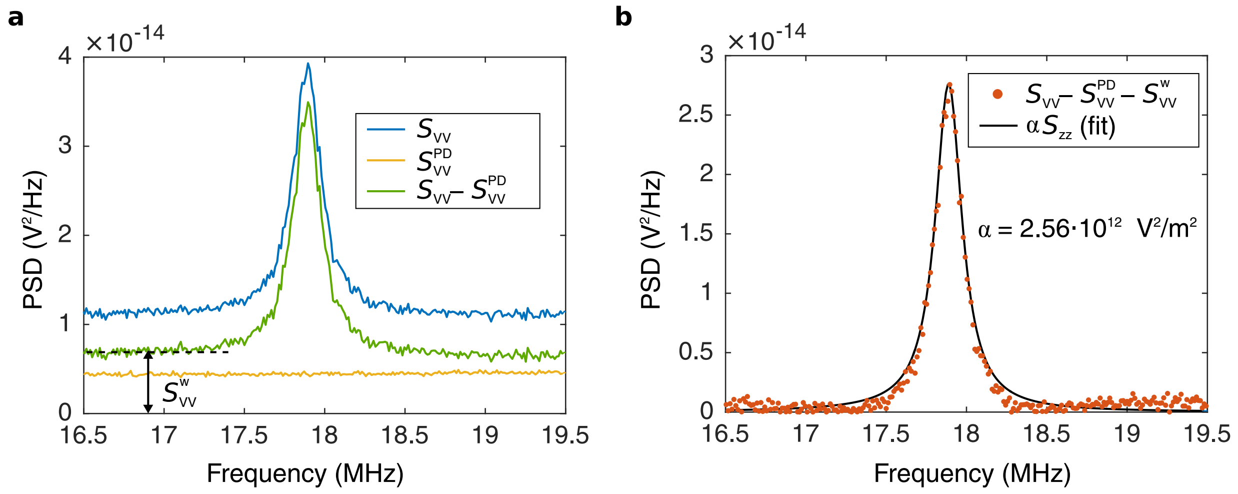

Supplementary Figure 4. **Calibration procedure**. (a) Measured power spectral density (PSD) of the fundamental mode of a 5-nm thick MoS_2 drum. The blue curve is the measured PSD, the yellow curve is the PSD of the photodiode dark current and the green curve is the resulting subtraction of $S_{\mathrm{VV}}(f) - S_{\mathrm{VV}}^{\mathrm{PD}}(f)$. The constant offset of the green curve accounts for all other sources of (white) noise in the system $S_{\mathrm{VV}}^{\mathrm{w}}$. (b) PSD of the motion of the drum, after subtracting the noise floor from the green curve in (a). The black curve is the fit of the PSD of the drum, from which the transduction factor α can be obtained

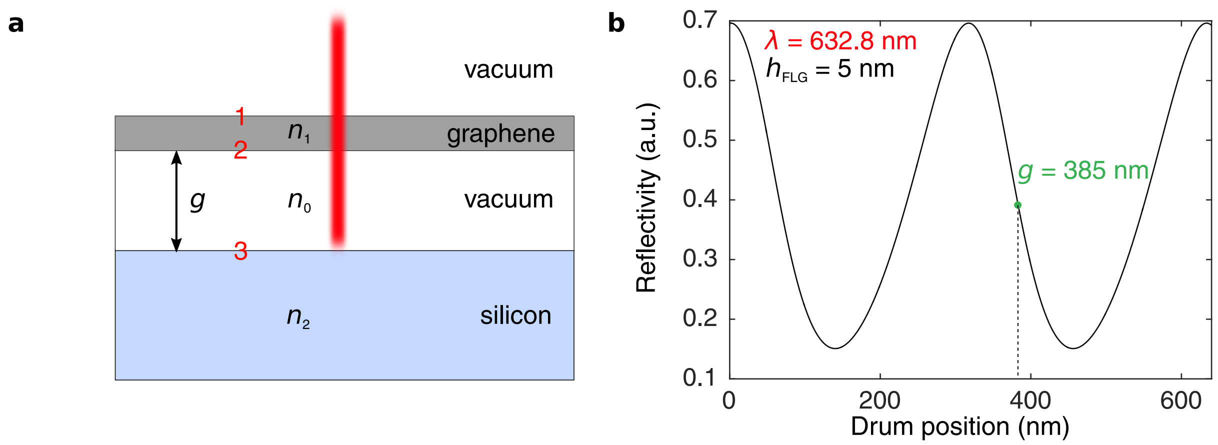

Supplementary Figure 5. **A schematic of the optical cavity and a model of the reflectivity of the cavity as a function of drum position**. (a) A schematic of the optical cavity. n_i are the refractive indices of medium i, g is the cavity depth and the numbers 1-3 represent the interfaces in the system. (b) Calculated reflectivity for a 5-nm thick graphene drum as a function of the position of the drum. The calculation is based on a measurement using a $\lambda = 632.8$ nm laser. The green dot represents the initial position of the drum (cavity depth) $g = 385$ nm.

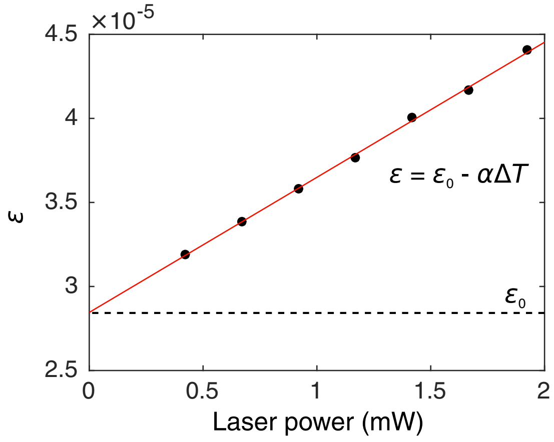

Supplementary Figure 6. **Temperature estimation**. Extracted strain (ε) as a function of the measurement laser power. The black dots are measured pre-strain calculated from the resonance frequency. The red line is a linear fit to the data. The black dashed line is the extrapolated initial strain (ε_0).

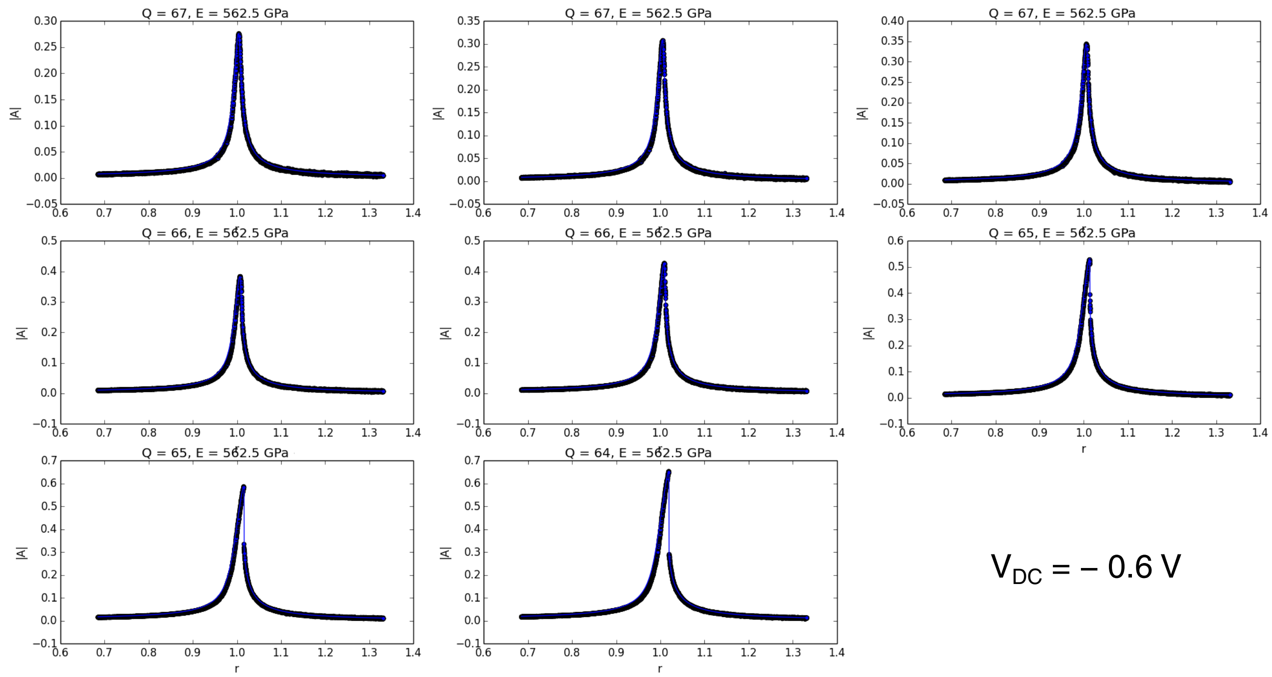

Supplementary Figure 7. **Normalized frequency vs. normalized amplitude of the nonlinear dynamic response of the drum at V_{dc} = -0.6 V**. Each of the panels represents a measurement at a different driving voltage V_{ac} (increasing from left to right). The black dots are the raw data and the blue lines represent the fits.

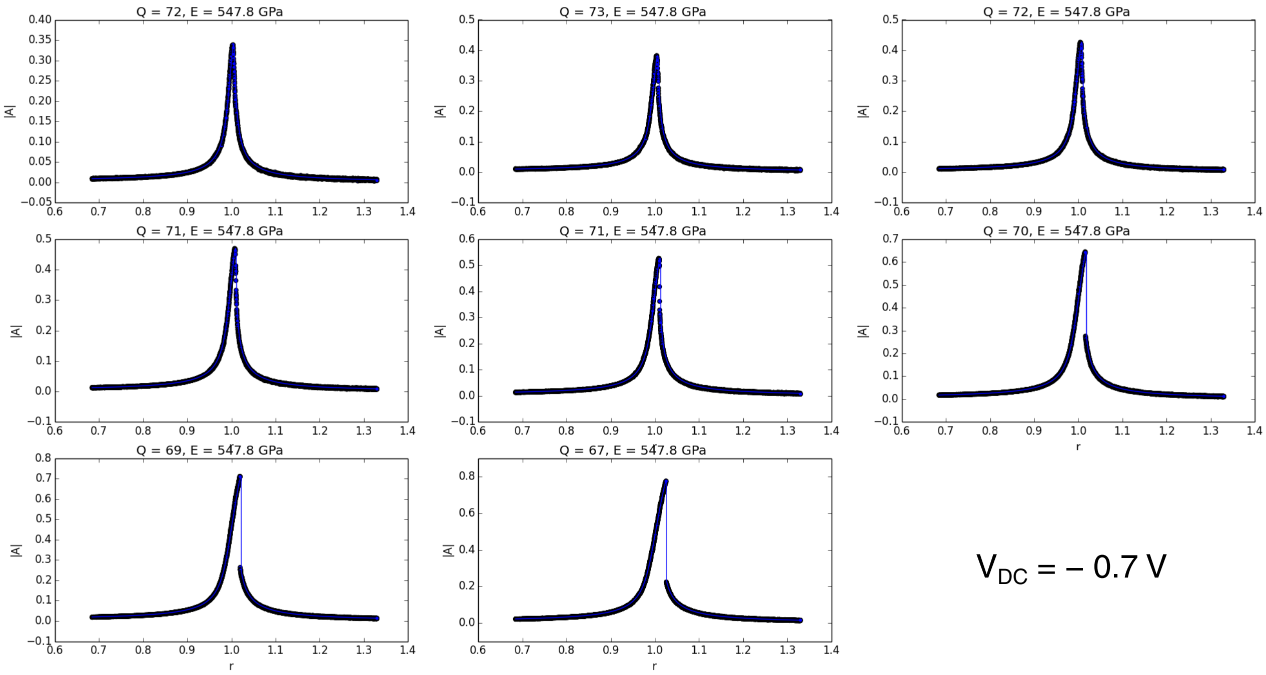

Supplementary Figure 8. **Normalized frequency vs. normalized amplitude of the nonlinear dynamic response of the drum at V_{dc} = -0.7 V**. Each of the panels represents a measurement at a different driving voltage V_{ac} (increasing from left to right). The black dots are the raw data and the blue lines represent the fits.

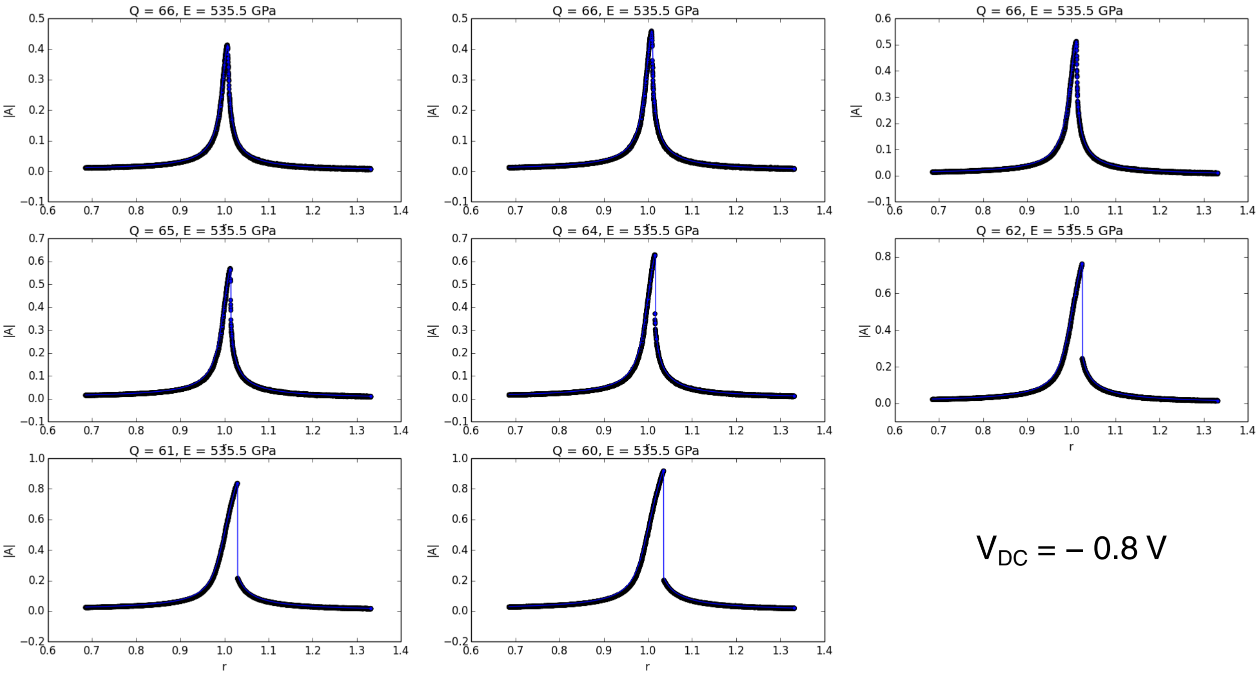

Supplementary Figure 9. **Normalized frequency vs. normalized amplitude of the nonlinear dynamic response of the drum at V_{dc} = -0.8 V**. Each of the panels represents a measurement at a different driving voltage V_{ac} (increasing from left to right). The black dots are the raw data and the blue lines represent the fits.

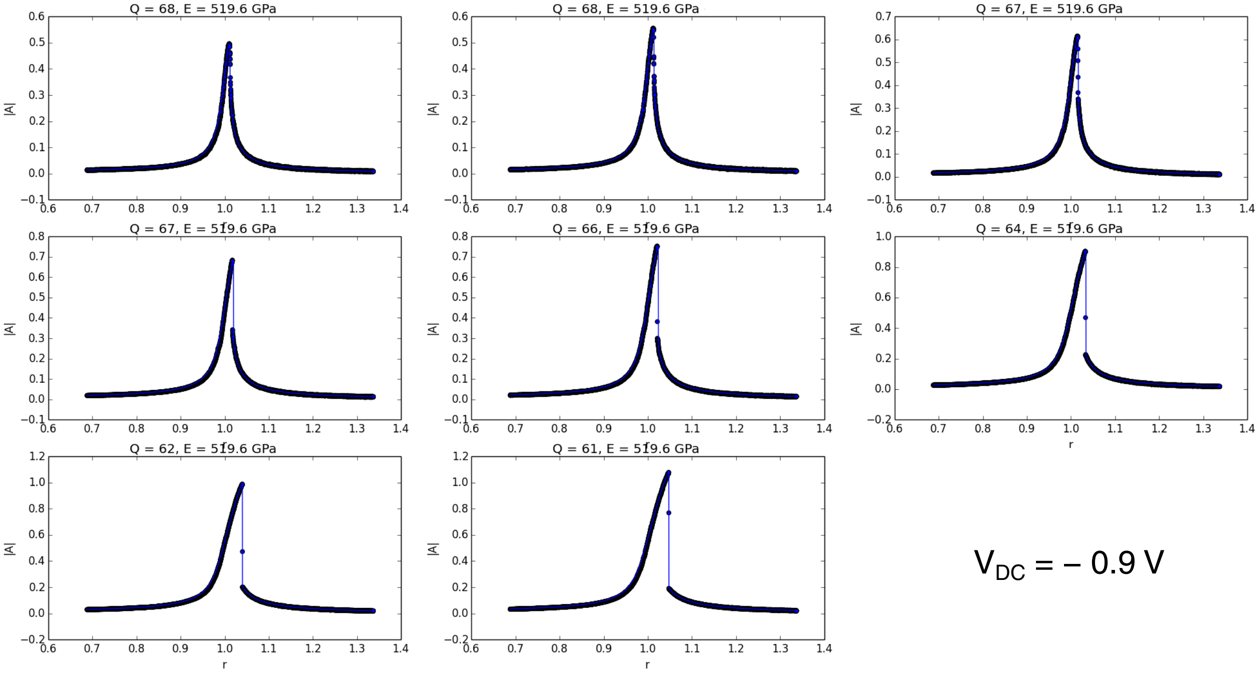

Supplementary Figure 10. **Normalized frequency vs. normalized amplitude of the nonlinear dynamic response of the drum at V_{dc} = -0.9 V**. Each of the panels represents a measurement at a different driving voltage V_{ac} (increasing from left to right). The black dots are the raw data and the blue lines represent the fits.

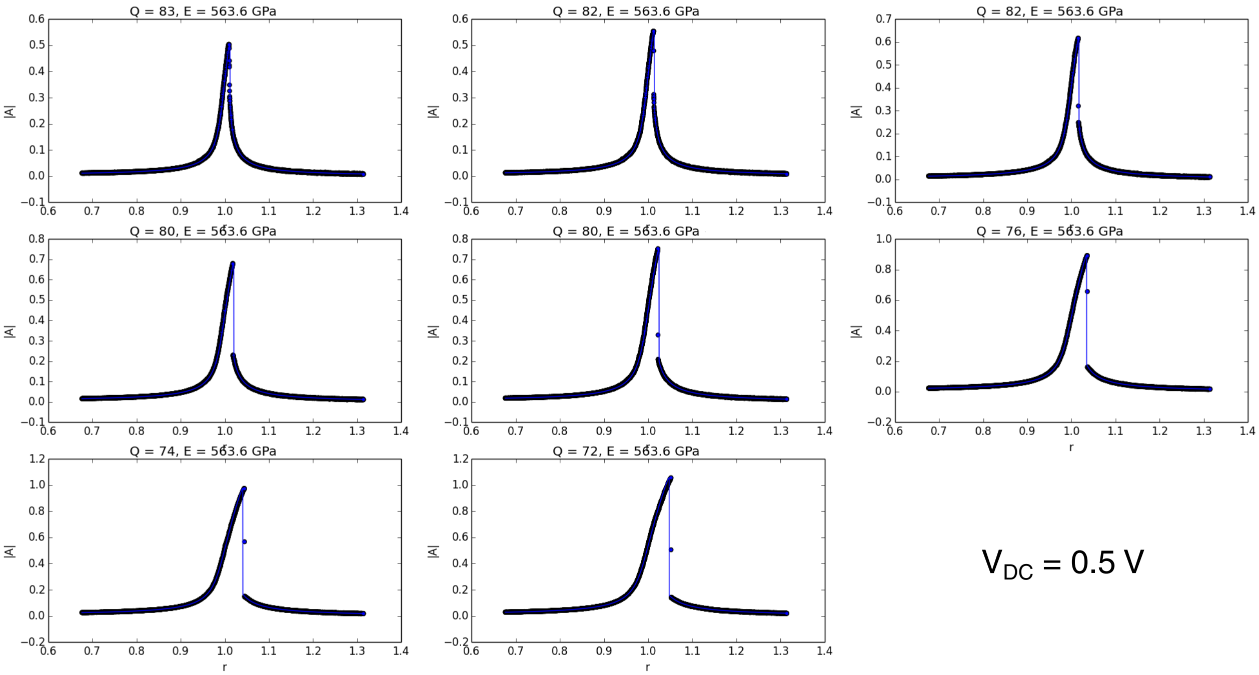

Supplementary Figure 11. **Normalized frequency vs. normalized amplitude of the nonlinear dynamic response of the drum at $V_{\mathrm{dc}} = 0.5$ V**. Each of the panels represents a measurement at a different driving voltage V_{ac} (increasing from left to right). The black dots are the raw data and the blue lines represent the fits.

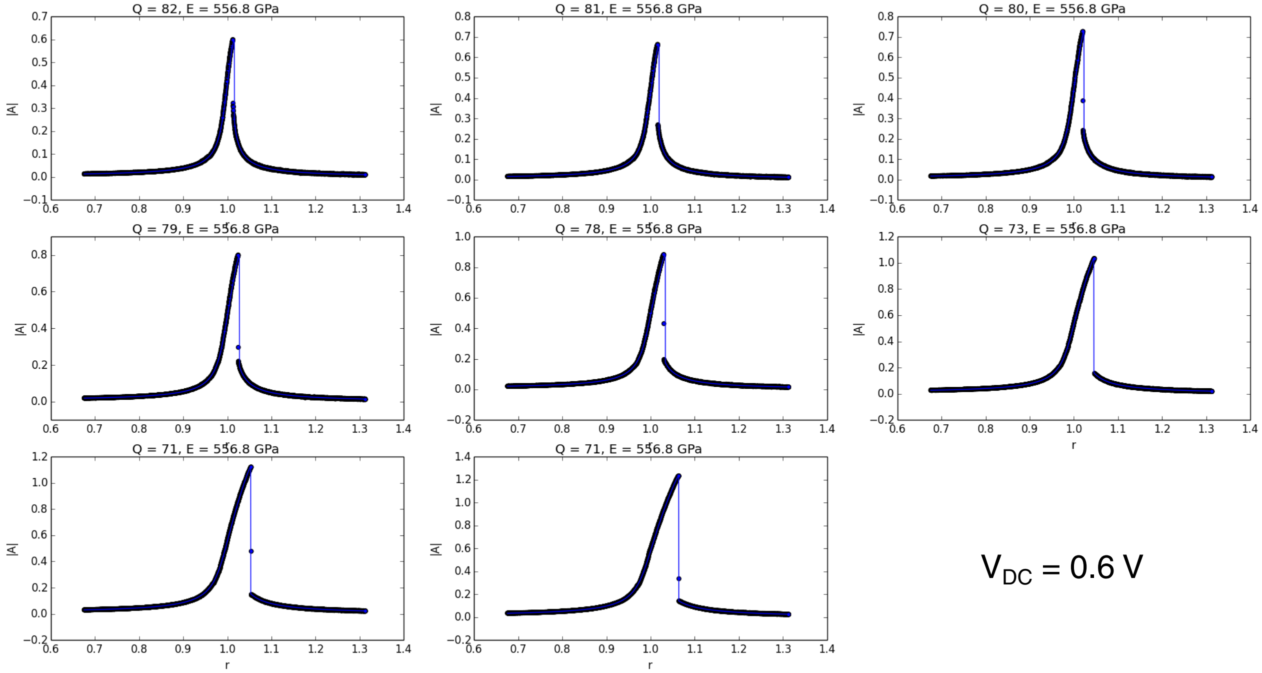

Supplementary Figure 12. **Normalized frequency vs. normalized amplitude of the nonlinear dynamic response of the drum at V_{dc} = 0.6 V**. Each of the panels represents a measurement at a different driving voltage V_{ac} (increasing from left to right). The black dots are the raw data and the blue lines represent the fits.

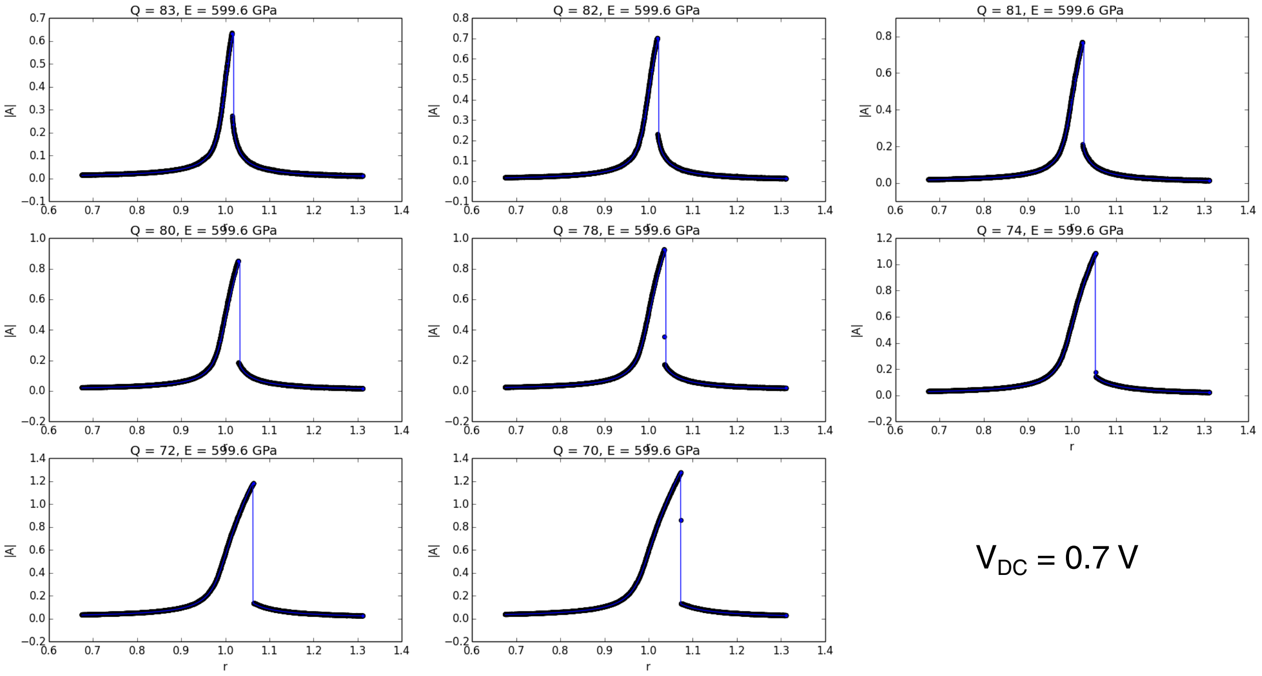

Supplementary Figure 13. **Normalized frequency vs. normalized amplitude of the nonlinear dynamic response of the drum at $V_{\rm dc}$ = 0.7 V**. Each of the panels represents a measurement at a different driving voltage $V_{\rm ac}$ (increasing from left to right). The black dots are the raw data and the blue lines represent the fits.

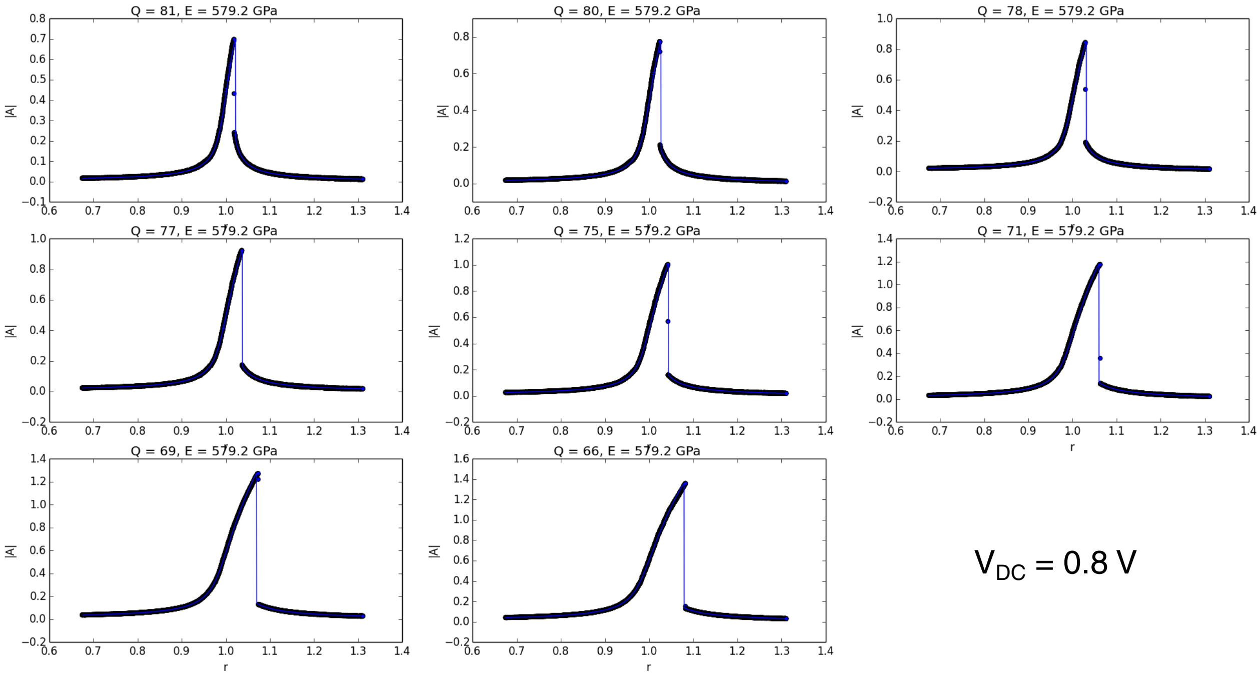

Supplementary Figure 14. **Normalized frequency vs. normalized amplitude of the nonlinear dynamic response of the drum at $V_{\rm dc}$ = 0.8 V**. Each of the panels represents a measurement at a different driving voltage $V_{\rm ac}$ (increasing from left to right). The black dots are the raw data and the blue lines represent the fits.

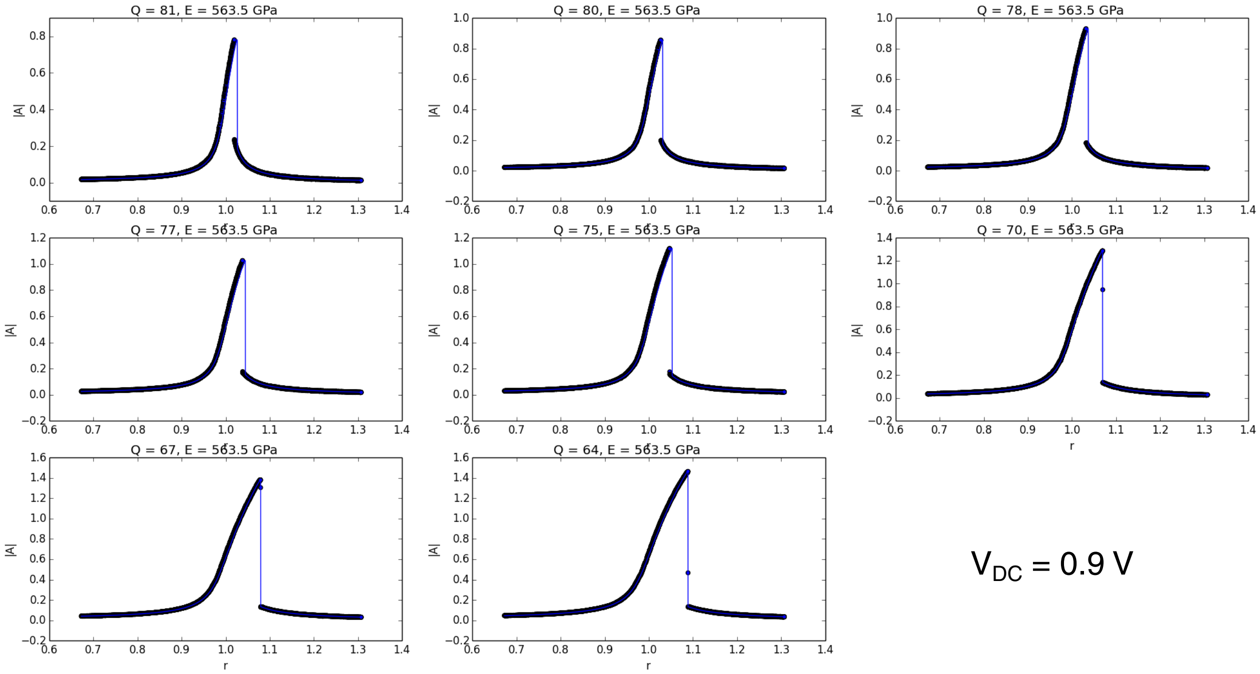

Supplementary Figure 15. **Normalized frequency vs. normalized amplitude of the nonlinear dynamic response of the drum at $V_{\mathrm{dc}} = 0.9$ V**. Each of the panels represents a measurement at a different driving voltage V_{ac} (increasing from left to right). The black dots are the raw data and the blue lines represent the fits.

Supplementary Note 1: Equations of motion

In this section the mathematical derivation of equations (1) and (3) of the main text is presented. The strain energy of the circular membrane can be obtained as [1]

$$U = \int_0^{2\pi}\int_0^R \frac{Eh}{2(1-\nu^2)}\Big(\epsilon_{rr}^2 + \epsilon_{\theta\theta}^2 + 2\nu\epsilon_{rr}\epsilon_{\theta\theta} + \frac{1-\nu}{2}\gamma_{r\theta}^2\Big) r\mathrm{d}r\mathrm{d}\theta, \tag{1}$$

where E is the Young's modulus, ν is the Poisson's ratio, h is the thickness and R is the radius of the membrane. Moreover, ϵ_{rr}, $\epsilon_{\theta\theta}$, and $\gamma_{r\theta}$ are the normal and shear strains that are determined as

$$\epsilon_{rr} = \frac{\partial u}{\partial r} + \frac{1}{2}\Big(\frac{\partial w}{\partial r}\Big)^2, \tag{2}$$

$$\epsilon_{\theta\theta} = \frac{\partial v}{r\partial\theta} + \frac{u}{r} + \frac{1}{2}\Big(\frac{\partial w}{r\partial\theta}\Big)^2, \tag{3}$$

$$\gamma_{r\theta} = \frac{\partial v}{\partial r} - \frac{v}{r} + \frac{\partial u}{r\partial\theta} + \Big(\frac{\partial w}{\partial r}\Big)\Big(\frac{\partial w}{r\partial\theta}\Big), \tag{4}$$

where u, v and w are the radial, tangential and transverse displacements respectively. For a membrane with fixed edges u and w shall vanish at $r = R$. Moreover, u should be zero at $r = 0$ for continuity and symmetry. Assuming only axisymmetric vibrations ($v = 0$ and $\partial w/\partial\theta = \partial v/\partial\theta = \partial u/\partial\theta = 0$) and fixed edges, the solution is approximated as [2]

$$w = x(t)J_0\Big(\alpha_0\frac{r}{R}\Big), \tag{5a}$$

$$u = u_0 r + r(R-r)\sum_{k=1}^{\bar{N}} q_k(t)r^{k-1}. \tag{5b}$$

Here it should be noted that for axisymmetric vibrations the shear strain $\gamma_{r\theta}$ would become zero. In Supplementary Equations (5a,b), $x(t)$ is the generalized coordinate associated with the transverse motion of the fundamental axisymmetric mode and $q_k(t)$ are the generalized coordinates associated with the radial motion. Moreover, J_0 is the Bessel function of order zero, and $\alpha_0 = 2.40483$. In addition, $\bar{N}$ is the number of terms in the expansion of radial displacement, and u_0 is the initial displacement due to pre-tension n_0 that is obtained from the initial stress $\sigma_0 = n_0/h$ as follows:

$$u_0 = \frac{\sigma_0(1-\nu)}{E}. \tag{6}$$

The kinetic energy of the membrane neglecting radial (i.e. in-plane) inertia , is given by

$$T = \frac{1}{2}\rho h \int_0^{2\pi}\int_0^R \dot{w}^2 r \mathrm{d}r\mathrm{d}\theta, \tag{7}$$

where the overdot indicates differentiation with respect to time t.

In the presence of transverse harmonic distributed pressure ($p = F_{\mathrm{el}}\cos(\omega t)/R^2\pi$) of constant direction, the virtual work done is

$$W = 2\pi\int_0^R p w r \mathrm{d}r = \frac{2}{R^2}\int_0^R F_{\mathrm{el}}\cos(\omega t) w r \mathrm{d}r, \tag{8}$$

where ω is the excitation frequency and F_{el} gives the force amplitude, positive in transverse direction. Higher-order terms in w are neglected in Supplementary Equation (8) [3]. The Lagrange equations of motion are

$$\frac{\mathrm{d}}{\mathrm{d}t}(\frac{\partial T}{\partial \dot{\mathbf{q}}}) - \frac{\partial T}{\partial \mathbf{q}} + \frac{\partial U}{\partial \mathbf{q}} = \frac{\partial W}{\partial \mathbf{q}}, \tag{9}$$

and $\mathbf{q} = [x(t), q_k(t)], k = 1, ..., \bar{N}$, is the vector including all the generalized coordinates.

Since radial inertia has been neglected, Supplementary Equation (9) leads to a system of nonlinear equations comprising of a single differential equation associated with the generalized coordinate $x(t)$ and $\bar{N}$ algebraic equations in terms of $q_k(t)$. By solving the $\bar{N}$ algebraic equations it is possible to determine $q_k(t)$ in terms of $x(t)$. This will reduce the $\bar{N}+1$ set of nonlinear equations to a single Duffing oscillator as follows:

$$m_{\mathrm{eff}}\ddot{x} + c\dot{x} + k_1 x + k_3 x^3 = \xi F_{el}\cos(\omega t), \tag{10}$$

where it is found that

$$m_{\mathrm{eff}} = 0.2695\, m,\ k_1 = 4.897 n_0,\ \xi = 0.432, \tag{11}$$

and c is the damping coefficient that has been added to the equation of motion to introduce linear viscous dissipation. The coefficients of m_{eff}, k_1 and the value of ξ are obtained from the numerical solution of Supplementary Equation (9) using Supplementary Equations (5a) and (5b) and are dependent on the deformation shape, i.e. the vibrational mode shape. Moreover, k_3 is the cubic stiffness, which is a function of the Young's modulus and Poisson's ratio, and its convergence and accuracy is determined by using different number of terms in the expression of the radial displacement (Supplementary Equation (5b)). The value of k_3

converges for $\bar{N} > 3$ and its relation to the Young's modulus can be determined by fixing the value of the Poisson's ratio and numerically solving the set of $\bar{N}$ Lagrange equations. k_3 can be expressed in the form:

$$k_3 = C_3(\nu)\frac{Eh\pi}{R^2}, \tag{12}$$

where C_3 is dimensionless constant which is a function of the Poisson's ratio. The solutions for C_3 as a function of ν are plotted in Supplementary Figure 1 for values of the Poisson's ratio between 0 and 0.35.

The relation between C_3 and ν is best described with the inverse of a second-order polynomial, namely:

$$C_3 = \frac{1}{1.269 - 0.967\nu - 0.269\nu^2}. \tag{13}$$

This functional dependence is similar to the one used for AFM nanoindentation measurements, often referred to as $q(\nu)$ [4].

Next, the following dimensionless parameters are introduced:

$$\hat{t} = \omega t, \tag{14a}$$

$$\hat{x} = x/h. \tag{14b}$$

By using Supplementary Equations (10) and (14) the following dimensionless equation of motion can be obtained:

$$r^2\ddot{\hat{x}} + \frac{1}{Q}r\dot{\hat{x}} + \hat{x} + \eta_3\hat{x}^3 = \lambda\cos(t), \tag{15}$$

where

$$\omega_0 = \sqrt{\frac{k_1}{m}}, Q = \frac{m\omega_0}{c}, \eta_3 = \frac{k_3h^2}{k_1}, \lambda = \frac{\xi F_{el}}{m\omega_0^2 h}, r = \frac{\omega}{\omega_0}. \tag{16}$$

Supplementary Equation (15) is valid for studying nonlinear vibrations of membranes subjected to external harmonic excitation in the frequency neighbourhood of the fundamental mode if the fundamental mode of vibration is not involved in an internal resonance with other modes. If such condition retains, then other modes accidentally excited will decay with time to zero due to the presence of damping [5]. In this work, it is assumed that this condition is preserved and therefore the response of the membrane is described by a single Duffing oscillator for performing nonlinear parameter estimation.

Supplementary Note 2: Nonlinear identification

In order to obtain the coefficients of the Duffing oscillator (Supplementary Equation (10)) from the measured nonlinear response curves, here we utilize the harmonic balance method. This method is a suitable and accurate mathematical technique that entails the solution of nonlinear equations to be approximated by a truncated Fourier series. In case of the dimensionless Duffing oscillator (i.e. Supplementary Equation (15)), a first order truncation has been shown to provide accurate results [5]. Hence,

$$\hat{x} \approx x_1 \sin t + x_2 \cos t. \tag{17}$$

Substituting Supplementary Equation (17) into Supplementary Equation (15) yields:

$$x_1(1-r^2) - r\frac{1}{Q}x_2 + \frac{3}{4}\eta_3 x_1 A^2 = 0, \tag{18a}$$

$$x_2(1-r^2) + r\frac{1}{Q}x_1 + \frac{3}{4}\eta_3 x_2 A^2 = \lambda, \tag{18b}$$

where $A = \sqrt{x_1^2 + x_2^2}$ is the amplitude of motion. Moreover, $x_1 = A\sin\phi$ and $x_2 = A\cos\phi$, ϕ being the phase difference between the excitation and the response. From Supplementary Equations (18a) and (18b) the following analytic frequency-amplitude relation could be found:

$$A^2\left[\left((1-r^2) + \frac{3}{4}\eta_3 A^2\right)^2 + (\frac{r}{Q})^2\right] = \lambda^2. \tag{19}$$

The idea of harmonic balance based parameter estimation is to follow a reverse path [6]. In other words, the identification is conducted by assuming that the vibration amplitude A and frequency ratio r are already known for every frequency step from experiments. Therefore, in order to obtain unknown parameters, the following system should be solved for every jth frequency step, $r^{(j)}$:

$$\begin{pmatrix} -r^{(j)}x_2 & \frac{3}{4}x_1A^2 \\ r^{(j)}x_1 & \frac{3}{4}x_2A^2 \end{pmatrix} \cdot \begin{bmatrix} \frac{1}{Q} \\ \eta_3 \end{bmatrix} = \begin{bmatrix} -x_1(1-(r^{(j)})^2) \\ -x_2(1-(r^{(j)})^2) + \lambda \end{bmatrix} \quad ,j = [1:m] \tag{20}$$

System (20) can be compactly written as $\bar{A_h} \cdot Y = \bar{B_h}$. This system is over constrained since it contains $2 \times m$ equations. In order to solve (20) and to estimate system parameters, least squares technique is used and the norm of the error $Er = \left(\bar{A_h} \cdot Y - \bar{B_h}\right) \cdot \left(\bar{A_h} \cdot Y - \bar{B_h}\right)^T$

should be minimized. Accordingly, here the pseudo-inverse of matrix A_h is calculated and the solution is obtained as follows:

$$Y = \left(\bar{A_h}^T \bar{A_h} \right)^{-1} \bar{A_h} \cdot \bar{B_h}. \tag{21}$$

A problem in utilizing the least squares technique is that the identified peak amplitudes in the frequency-response curves do not correspond to the ones obtained from the experiments. In order to resolve this issue, a correction on the quality factor is made by making use of the following expression (see [6] for the derivation details):

$$Q = \frac{1}{2} \left[\sqrt{\frac{1}{2} + \frac{3}{8}\eta_3 A_{\max}^2 - \sqrt{(\frac{1}{2} + \frac{3}{8}\eta_3 A_{\max}^2)^2 - \frac{\lambda^2}{4A_{\max}^2}}} \right]^{-1}, \tag{22}$$

in which $A_{\max}$ is the experimentally measured peak amplitude for each frequency-amplitude curve. This will yield the nonlinear identification procedure to a single fit parameter algorithm for the estimation of η_3.

Supplementary Note 3: Estimation of the electrostatic spring softening

In this section the effect of nonlinearities in the electrostatic force on the nonlinear spring constant is estimated.

The electrostatic force acting on the membrane is given by

$$F_{\mathrm{el}} = -\frac{\mathrm{d}U_{\mathrm{el}}}{\mathrm{d}x} = -\frac{1}{2}\frac{\mathrm{d}C_{\mathrm{g}}}{\mathrm{d}x}V^2, \tag{23}$$

where $U_{\mathrm{el}} = \frac{1}{2}C_{\mathrm{g}}V^2$ is the electrostatic energy, $V = V_{\mathrm{dc}} + V_{\mathrm{ac}}\cos(\omega t)$ is the applied voltage and C_{g} is the gate capacitance. Assuming $x << g$, where g is the gap between the membrane and the backgate, the gate capacitance can be approximated using a parallel plate capacitor model:

$$C_{\mathrm{g}} = \varepsilon_0 \frac{R^2\pi}{g-x}, \tag{24}$$

where R is the radius of the membrane and ε_0 is the vacuum permittivity. The resulting electrostatic force is given by

$$F = \frac{1}{2}\frac{\varepsilon_0 R^2\pi}{(g-x)^2}(V_{\mathrm{dc}} + V_{\mathrm{ac}}\cos(\omega t))^2. \tag{25}$$

If we expand this expression around $x = 0$, using $x/g << 1$, we get

$$F \approx \frac{1}{2}\varepsilon_0 R^2\pi(V_{\mathrm{dc}} + V_{\mathrm{ac}}\cos(\omega t))^2\Big[\frac{1}{g^2} + \frac{2x}{g^3} + \frac{3x^2}{g^4} + \frac{4x^3}{g^5}\Big]. \tag{26}$$

The first term ($\frac{1}{g^2}$) is the dominant electrostatic actuation term and the second term is what is usually described as a spring softening term ($\frac{2x}{g^3}$). This term influences only the linear spring constant of the resonator. The term including x^3 will have a softening effect on the cubic spring constant: $k_{3,\mathrm{soft}} = \frac{2}{g^5}\varepsilon_0 R^2\pi V_{\mathrm{dc}}^2$ (for $V_{\mathrm{dc}} >> V_{\mathrm{ac}}$ and $g >> x$). The resulting cubic spring constant $k_{3,\mathrm{tot}}$ will be given by

$$k_{3,\mathrm{tot}} = k_3 - k_{3,\mathrm{soft}}. \tag{27}$$

The ratio of the two contributions (using the expression for k_3 from Supplementary Equation (12)) is

$$\frac{k_3}{k_{3,\text{soft}}} = \frac{1}{1.269 - 0.967\nu - 0.269\nu^2} \frac{Ehg^5}{2\varepsilon_0 R^4 V_{\text{dc}}^2}. \tag{28}$$

For a Young's modulus $E = 594$ GPa, radius of $R = 2.5\ \mu m$, thickness of $h = 5$ nm, gap size $g = 385$ nm and $V_{\text{dc}} \leq 3$ V, this ratio becomes

$$\frac{k_3}{k_{3,\text{soft}}} \approx 3000, \tag{29}$$

which means that the electrostatic softening will have a negligible effect on the extracted Young's modulus (resulting in an error of $< 0.1\%$). It should be noted that the cavity depth has a significant influence on the effect of electrostatic softening of the cubic spring constant. To get reasonable error margins ($< 5\%$), the ratio of Supplementary Equation (29) should be kept above 20.

Supplementary Note 4: The effect of the Young's modulus on the strength of the nonlinear dynamic response

In Supplementary Figure 2 we show the frequency response of a strongly-driven graphene drum (black dots) under a constant force ($F_{\mathrm{RMS}} = 48$ pN). The coloured curves are the modelled response curves under constant force and with a fixed quality factor ($Q = 129$) and resonance frequency $f_0 = 14.7$ MHz). The different colours correspond to the frequency responses of the model using different values for the Young's modulus to show how the nonlinear response is influenced by the Young's modulus.

Supplementary Note 5: Determination of the driving force

In this section the electrostatic force is determined by two methods: (i) based on the geometry and the voltage applied on the drum and (ii) based on the measured amplitudes at resonance.

The electrostatic force acting on the membrane at a frequency ω, assuming a parallel plate capacitor model, can be calculated as:

$$F_{\mathrm{calc}}(\omega) = \xi \frac{\varepsilon_0 R^2 \pi}{2g^2} (V_{\mathrm{dc}} + V_{\mathrm{ac}} \cos(\omega t))^2 = \xi \frac{\varepsilon_0 R^2 \pi}{2g^2} \left[V_{\mathrm{dc}}^2 + \frac{1}{2} V_{\mathrm{ac}}^2 + 2 V_{\mathrm{dc}} V_{\mathrm{ac}} \cos(\omega t) + \frac{1}{2} V_{\mathrm{ac}}^2 \cos(2\omega t) \right], \tag{30}$$

where g is the gap size and $\xi = 0.432$ is a correction factor that accounts for the fundamental mode shape of a fixed circular membrane (see Section 1 and main text).

In a homodyne detection scheme, only the motion resulting from a force at frequency ω can be detected. Therefore, the effective force on the drum will be:

$$F_{\mathrm{calc}}(\omega) = \xi \frac{\varepsilon_0 R^2 \pi}{2g^2} \left[2 V_{\mathrm{dc}} V_{\mathrm{ac}} \cos(\omega t) \right]. \tag{31}$$

It is worth mentioning that due to the fact that the output of the VNA has 50Ω impedance and the drum is a capacitor (high impedance), the ac voltage felt by the drum is double the voltage that is output by the VNA ($V_{\mathrm{ac}} = 2V_{\mathrm{ac,VNA}}$).

Knowing the applied driving force is crucial for fitting the nonlinear response curves and finding the Young's modulus of the membranes, and it therefore needs to be determined very accurately. Even though it can be easily calculated using Supplementary Equation (30), there are three main sources of uncertainty that can alter the calculated value. The first one is the gap size g. Even though the gap size is fixed by the sample fabrication and can be easily measured, the actual position of the drum due to membrane slack and imperfections is unknown. This can change the effective gap size, and therefore the effective force by up to 5 % (assuming slack of 20 nm over a 385 nm gap). A second source of uncertainty is the dc voltage felt by the drum. It is well known from resonance frequency measurements that the resonance frequency minimum in f_0 vs. V_{dc} curves is often shifted away from the so-called charge neutrality point (CNP) [7, 8]. Similar effects have been observed in carbon nanotubes and are often attributed to trapped charges [7, 9, 10]. These charges manifest as

an added dc voltage of the drum, and can significantly affect the effective electrostatic force. Finally, due to the presence of the AuPd electrode underneath the drum, the electric field will be screened by the edges of the electrode around the perimeter of the drum. This will lower the effective capacitance of the drum and therefore also the effective electrostatic force felt by it. For a flat drum, using a gap size of 385 nm and a 100 nm-thick AuPd electrode, such fringe field effects can account for up to 15 % of overestimation of the force.

To correct for these effects, we calculate the expected peak amplitude at resonance ($x_{\mathrm{calc,RMS}}|_{\omega=\omega_0}$) using the force calculated using Supplementary Equation (31). In the linear vibration regime, this function is expected to be linear with the force, hence with the applied ac voltage:

$$x_{\mathrm{calc,RMS}}|_{\omega=\omega_0} = \frac{Q}{\omega_0^2 m_{\mathrm{eff}}} F_{\mathrm{calc,RMS}}. \tag{32}$$

$$x_{\mathrm{calc,RMS}}|_{\omega=\omega_0} = \frac{Q}{\omega_0^2 m_{\mathrm{eff}}} \xi \frac{\varepsilon_0 R^2 \pi}{g^2} V_{\mathrm{dc}} V_{\mathrm{ac,RMS}} = \beta V_{\mathrm{ac}}. \tag{33}$$

The slope of the x_{RMS} vs. $V_{\mathrm{ac,RMS}}$ curve at low ac voltages (linear regime) will be a constant β, defined as:

$$\beta = \frac{\mathrm{d}x_{\mathrm{calc,RMS}}|_{\omega=\omega_0}}{\mathrm{d}V_{\mathrm{ac,RMS}}} = \frac{Q}{\omega_0^2 m_{\mathrm{eff}}} \xi \frac{\varepsilon_0 R^2 \pi}{g^2} V_{\mathrm{dc}}; \tag{34}$$

$$\beta = \frac{Q}{\omega_0^2 m_{\mathrm{eff}} V_{\mathrm{ac,RMS}}} F_{\mathrm{calc,RMS}}. \tag{35}$$

Knowing β, we can calculate the force for any driving ac voltage:

$$F_{\mathrm{calc,RMS}} = \beta \frac{\omega_0^2 m_{\mathrm{eff}}}{Q} V_{\mathrm{ac,RMS}}. \tag{36}$$

The actual force felt by the drum (F_{RMS}) will be modified depending on the uncertainties in determining V_{dc} (V'_{dc}) and g (g'), possibly resulting in a different slope of the x_{RMS} vs. $V_{\mathrm{ac,RMS}}$ curve: β'. We can use this value to cross-check and correct for the actual force (F_{RMS}):

$$F_{\mathrm{RMS}} = \beta' \frac{\omega_0^2 m_{\mathrm{eff}}}{Q} V_{\mathrm{ac,RMS}}. \tag{37}$$

To visualize the procedure, in Supplementary Figure 3, we present data from a 4-μm in diameter, 8-nm thick FLG drum. The calibrated amplitude at resonance (or $x_{\mathrm{RMS,max}}$ in the nonlinear regime) is plotted as blue dots (similar data can be seen in [11]). The calculated expected amplitude based on Supplementary Equation (33) is represented by the red line. To correct for the difference in slopes in the linear part (left hand side), we fit the slope of the measured amplitude to extract β'. The resulting error in the force estimation is roughly 10 %, which, assuming that the error is due to trapped charges, translates to a 0.1 V offset over the applied $V_{\mathrm{dc}} = 1$ V. This offset can easily stem from residual charges on the graphene flake [7].

As long as the force scales linearly with the applied driving voltage (and there are no significant nonlinearities in the actuation), this method can be easily extended to calibrate the force of other actuation methods, like optothermal actuation, since the force can be extracted from the calibrated amplitude data. This is provided that the Q-factor is known and the displacement x can be properly calibrated.

Supplementary Note 6: Calibration of the amplitude

In this section the procedure of calibration of the motion amplitude using the resonator's Brownian noise is explained. In addition, the effect of nonlinearities in the readout method on the calibration is estimated.

To convert the measured signal (in Volts) to the motion amplitude of the resonator (in metres), we use thermal calibration (we follow closely the procedure described in [12]). This means that we can relate the time-averaged undriven (Brownian) motion of the drum to its thermal energy. Using the equipartition theorem, the following relation between the drum's amplitude and its thermal noise power spectral density (PSD) can be stated:

$$\langle x_n^2(t) \rangle = \int_0^\infty \mathrm{d}f S_{\mathrm{zz}}(f), \quad (38)$$

where $\langle x_n^2(t) \rangle$ is the mean-square amplitude of motion of the n-th mode of the drum, f is the frequency, and $S_{\mathrm{zz}}(f)$ is the measured one-sided spectral density of the drum's motion.

The thermal noise PSD of the drum ($S_{\mathrm{zz}}(f)$) is given by [12]:

$$S_{\mathrm{zz}}(f) = \frac{k_B T f_n}{2\pi^3 m_{\mathrm{eff},n} Q_n[(f^2 - f_n^2)^2 + (f f_n/Q_n)^2]}, \quad (39)$$

where k_B is the Boltzmann constant, T is the temperature (in our case, we take $T = 293$ K), and f_n, $m_{\mathrm{eff},n}$ and Q_n are the resonance frequency, effective mass ($m_{\mathrm{eff}} = 0.2695\, m$ at the centre of the drum [12]) and quality factor of the n-th resonance mode respectively.

We start by experimentally measuring the PSD using a spectrum analyser, $S_{\mathrm{VV}}(f)$. This is obtained as $V(f)^2/\Delta f$, where $V(f)$ is the voltage spectrum of the resonator's motion, measured with a bandwidth Δf. The unit of the obtained signal is then V^2/Hz. $S_{\mathrm{VV}}(f)$ represents the total PSD of the system. This includes noise from various sources in the detection system, among which the dark current noise of the photodiode ($S_{\mathrm{VV}}^{\mathrm{PD}}$), which can be easily measured and eliminated. Assuming all other sources of noise to be white, we can consider them to be a flat additive contribution($S_{\mathrm{VV}}^{\mathrm{w}}$) to the total PSD. $S_{\mathrm{VV}}^{\mathrm{w}}$ also determines the noise floor of the measurement. The remaining signal can be entirely attributed to the thermal noise PSD of the drum ($S_{\mathrm{zz}}(f)$) multiplied by a transduction factor α (V^2/m^2), which depends on the mechanical-optical-electrical transduction of the system. It is worth mentioning that α is highly susceptible to changes in the measurement laser intensity, po-

sition and focus of the drum, the cavity depth and optical/electrical losses in the system. To ensure that the value of α stays constant throughout the measurements we take all measurements in one go, without changing the sample position.

We can now write a general relation between the aforementioned quantities:

$$S_{\mathrm{VV}}(f) = S_{\mathrm{VV}}^{\mathrm{w}} + S_{\mathrm{VV}}^{\mathrm{PD}}(f) + \alpha S_{\mathrm{zz}}(f) \tag{40}$$

In Supplementary Figure 4 we show an example of a calibration measurement of the MoS_2 drum shown in the main text. The measured total PSD of the drum (S_{VV}) is shown by the blue curve in Supplementary Figure 4 (a). Subtracting the photodiode dark current noise $S_{\mathrm{VV}}^{\mathrm{PD}}$ (yellow curve), we get the resulting green curve. This curve contains both $\alpha S_{\mathrm{zz}}(f)$ and a constant noise floor ($S_{\mathrm{VV}}^{\mathrm{w}}$), assuming a white noise background. Further subtracting this offset results in the data (red points) shown in Supplementary Figure 4 (b). This curve can now be fitted using Supplementary Equation (39) multiplied by the transduction factor α. This fit is shown by the black curve in Supplementary Figure 4 (b). From this fit, we can determine 3 fit parameters: the resonance frequency f_0, the quality factor Q and, most importantly, the transduction factor α (in this case $\alpha = 2.56 \cdot 10^{12}\,\mathrm{V}^2/\mathrm{m}^2$).

Using this transduction factor, we can now determine the amplitude (in nanometres) of the driven motion as well. The magnitude of the driven motion at resonance is obtained from a two-port network analyser measurement and is given by $|S_{21}(f_0)|$ $[V/V]$. Multiplying this by the RMS value of the ac driving voltage ($V_{\mathrm{ac,RMS}}$) gives us the RMS value of the time averaged motion amplitude of the drum in Volts ($A_{\mathrm{RMS}}(f)$)

$$A_{\mathrm{RMS}}(f) = \sqrt{\langle A^2(f)\rangle} = |S_{21}|(f) \cdot V_{\mathrm{ac,RMS}}. \tag{41}$$

This amplitude can be converted to metres ($x_{\mathrm{RMS}}(f)$) considering a constant transduction factor α by:

$$x_{\mathrm{RMS}}(f) = \sqrt{\langle x^2(f)\rangle} = \frac{1}{\sqrt{\alpha}} A_{\mathrm{RMS}}(f). \tag{42}$$

Supplementary Note 7: Calibration uncertainties

The entire calibration procedure is done assuming that the transduction factor α is constant, i.e. the reflectivity of the system depends linearly on the position of the drum. This can be understood if we look at the reflectivity of the system, which can be described using [13]:

$$R = |\frac{r_1 + r_2 e^{-i\delta_1} + r_3 e^{-i\delta_2} + r_1 r_2 r_3 e^{-i(\delta_1+\delta_2)}}{1 + r_1 r_2 e^{-i\delta_2} + r_1 r_3 e^{-i(\delta_1+\delta_2)} + r_2 r_3 e^{-i\delta_2}}|^2, \tag{43}$$

where r_1, r_2 and r_3 represent the reflections from interfaces 1-3 and δ_1 and δ_2 are the phases that the light acquires while travelling through the graphene and the cavity (vacuum) respectively (see Fig. 5 (a)). Fixing the thickness of the graphene and the laser wavelength, in Fig. 5 (b) we plot the reflectivity as a function of the position of the drum for a 5-nm thick graphene flake and a red laser with $\lambda = 632.8$ nm.

The transduction factor $\sqrt{\alpha}$ is in fact proportional to the derivative of this curve with respect to the drum position at the point $g = 385$ nm. Even though the cavity depth is adjusted such that we sit on the linear part of the curve, there are still higher order terms from the linearisation which can result in an error when determining the amplitude.

For thin flakes, the intensity of the reflected light can be approximated by:

$$I = A + B\cos(4\pi\frac{g+x}{\lambda}), \tag{44}$$

where I is the intensity of the light on the photodiode, A and B are constant coefficients, g is the cavity depth, λ is the wavelength of light, and x is the vertical displacement of the drum. This equation can be now expanded around the static position of the drum ($x = 0$):

$$I = A + B\cos\left(4\pi\frac{g}{\lambda}\right) - B\frac{4\pi}{\lambda}\sin\left(4\pi\frac{g}{\lambda}\right)x - B\left(\frac{4\pi}{\lambda}\right)^2\cos\left(4\pi\frac{g}{\lambda}\right)\frac{x^2}{2} + B\left(\frac{4\pi}{\lambda}\right)^3\sin\left(4\pi\frac{g}{\lambda}\right)\frac{x^3}{6}. \tag{45}$$

Assuming a periodic motion of the membrane, the amplitude is given by: $x = x_{\max}\cos\omega t$. Replacing this in Supplementary Equation (45) gives:

$$I = A + B\cos\left(4\pi\frac{g}{\lambda}\right) - B\frac{4\pi}{\lambda}\sin\left(4\pi\frac{g}{\lambda}\right)x_{\max}\cos\omega t - \frac{B}{2}\left(\frac{4\pi}{\lambda}\right)^2\cos\left(4\pi\frac{g}{\lambda}\right)x_{\max}^2\cos^2\omega t + \\ + \frac{B}{6}\left(\frac{4\pi}{\lambda}\right)^3\sin\left(4\pi\frac{g}{\lambda}\right)x_{\max}^3\cos^3\omega t. \tag{46}$$

Substituting $\gamma = 4\pi\frac{g}{\lambda}$, $C_0 = A + B\cos\left(4\pi\frac{g}{\lambda}\right)$, $C_1 = B\frac{4\pi}{\lambda}$, $C_2 = \frac{B}{2}\left(\frac{4\pi}{\lambda}\right)^2$ and $C_3 = \frac{B}{6}\left(\frac{4\pi}{\lambda}\right)^3$ we get:

$$I = C_0 - (C_1\sin\gamma)x_{\max}\cos\omega t - (C_2\cos\gamma)x_{\max}^2\cos^2\omega t + (C_3\sin\gamma)x_{\max}^3\cos^3\omega t. \tag{47}$$

$$I = \overbrace{C_0 - \frac{C_2\cos\gamma}{2}x_{\max}^2}^{0} + \overbrace{\left[\frac{3}{4}(C_3\sin\gamma)x_{\max}^3 - (C_1\sin\gamma)x_{\max}\right]}^{\text{f}}\cos\omega t - \\ - \underbrace{\frac{(C_2\cos\gamma)}{2}x_{\max}^2}_{\text{2f}}\cos 2\omega t + \underbrace{\frac{C_3\sin\gamma}{4}x_{\max}^3}_{\text{3f}}\cos 3\omega t. \tag{48}$$

Using homodyne detection with a very narrow bandwidth filter (in our case < 1kHz), we are only sensitive to the $\cos\omega t$ component of Supplementary Equation (48) (labeled f), hence the actual intensity detected by the VNA at the resonance frequency ω_0 is:

$$I(\omega) = \frac{3}{4}(C_3\sin\gamma)x_{\max}^3 - (C_1\sin\gamma)x_{\max}. \tag{49}$$

The linear coefficient of Supplementary Equation (49) corresponds to the transduction coefficient extracted using the calibration procedure $\sqrt{\alpha}$. At small amplitudes, the cubic component plays little role. The error that the cubic term introduces in the amplitude calibration at large amplitudes is therefore given by:

$$\varepsilon = \frac{\frac{3}{4}(C_3\sin\gamma)x_{\max}^3}{(C_1\sin\gamma)x_{\max}} = \frac{3}{4}\frac{C_3}{C_1}x_{\max}^2, \tag{50}$$

or:

$$\varepsilon = 2\frac{\pi^2}{\lambda^2}x_{\max}^2 \tag{51}$$

Hence, using a laser with $\lambda = 632.8$ nm, for amplitudes of up to $x_{\max} = 10$ nm, the error that we make in the estimation of the amplitude is $|\varepsilon| < 0.5$ %. We note that this error is independent of the the gap size g.

Another important parameter that may influence the calibration is the actual temperature of the membrane. Even though the measurements are conducted at room temperature, the measurement laser locally heats up the membrane. For this reason, the laser power throughout the measurement is kept relatively low (0.42 mW). To estimate the temperature increase due to the laser heating, we perform measurements at varying laser power. From the measured resonance frequency we can determine the strain of the membrane (ε). This measurement is shown in Supplementary Figure 6.

As expected, the strain grows linearly with the laser power, due to a finite temperature increase ΔT. This can be modelled with a simple equation:

$$\varepsilon = \varepsilon_0 - \alpha_T \Delta T \tag{52}$$

where ε_0 is the mechanical pre-strain, α_T is the thermal expansion coefficient and ΔT is the temperature increase. The second term in the equation is the thermally induced strain. Since α_T for graphene is negative [14], the total strain of the drum increases with increasing laser power.

By fitting the measured data with a linear fit, we can extrapolate the curve and estimate ε_0 (see Supplementary Figure 6). Using $\alpha_T \approx -6 \cdot 10^{-6} K^{-1}$, we find the temperature increase at 0.42 mW laser power to be less than 0.8 K. This temperature difference, following Supplementary Equations (39) and (42), results in about 0.1 % error in the amplitude calibration.

Supplementary References

[1] Amabili, M. *Nonlinear vibrations and stability of shells and plates* (Cambridge University Press, 2008).

[2] Timoshenko, S. & Woinowsky-Krieger, S. Theory of plates and shells (1959).

[3] Amabili, M. & Breslavsky, I. D. Displacement dependent pressure load for finite deflection of doubly-curved thick shells and plates. *International Journal of Non-Linear Mechanics* **77**, 265–273 (2015).

[4] Lee, C., Wei, X., Kysar, J. W. & Hone, J. Measurement of the elastic properties and intrinsic strength of monolayer graphene. *Science* **321**, 385–388 (2008).

[5] Nayfeh, A. H. & Mook, D. T. *Nonlinear oscillations* (John Wiley & Sons, 2008).

[6] Amabili, M., Alijani, F. & Delannoy, J. Damping for large-amplitude vibrations of plates and curved panels, part 2: Identification and comparisons. *International Journal of Non-Linear Mechanics* **85**, 226–240 (2016).

[7] Chen, C. *et al.* Performance of monolayer graphene nanomechanical resonators with electrical readout. *Nature Nanotechnology* **4**, 861–867 (2009).

[8] Davidovikj, D. *et al.* Visualizing the motion of graphene nanodrums. *Nano letters* **16**, 2768–2773 (2016).

[9] Sazonova, V. *et al.* A tunable carbon nanotube electromechanical oscillator. *Nature* **431**, 284–287 (2004).

[10] Witkamp, B., Poot, M. & van der Zant, H. S. Bending-mode vibration of a suspended nanotube resonator. *Nano letters* **6**, 2904–2908 (2006).

[11] Bunch, J. S. *et al.* Electromechanical resonators from graphene sheets. *Science* **315**, 490–493 (2007).

[12] Hauer, B., Doolin, C., Beach, K. & Davis, J. A general procedure for thermomechanical calibration of nano/micro-mechanical resonators. *Annals of Physics* **339**, 181–207 (2013).

[13] Blake, P. *et al.* Making graphene visible. *Applied Physics Letters* **91**, 063124 (2007).

[14] Singh, V. *et al.* Probing thermal expansion of graphene and modal dispersion at low-temperature usinggraphene nanoelectromechanical systems resonators. *Nanotechnology* **21**, 165204 (2010).
